# Short-term exposure to ambient air pollution and onset of severe mental disorders: a case-crossover study in Northwestern China

**DOI:** 10.1186/s12889-025-25906-z

**Published:** 2025-12-22

**Authors:** Yajin Han, Guangrui Yang, Jinshi Wang, Weimin Pan, Xiaofeng Luo

**Affiliations:** 1 School of Public Health, Lanzhou University, Gansu, China; 2 Department of Epidemiology and Biostatistics, School of Public Health, Shanghai Jiao Tong University School of Medicine, Shanghai, China; 3 Institute of Chronic Noncommunicable Disease Control and Prevention, Gansu Provincial Center for Disease Control and Prevention, 310 Donggang West Rd, Lanzhou, 730000 China; 4 School of Public Health, Medical Campus, Lanzhou University, Lanzhou, China Qinbo Building, 199 Donggang West Rd, 730000

**Keywords:** Mental disorders, Ambient air pollution, Case-crossover, Short-term exposure

## Abstract

**Background:**

The associations between short-term exposure to air pollution and severe mental disorders (SMDs) remain poorly understood, particularly in China and among potentially vulnerable subpopulations.

**Methods:**

We conducted a time-stratified case-crossover study of 49,707 individuals diagnosed with SMDs in Gansu Province, China, from 2013 to 2020. Individual-level exposures to particulate matter ≤ 2.5 μm in aerodynamic diameter (PM_2.5_), particulate matter ≤ 10 μm in aerodynamic diameter (PM_10_), sulfur dioxide (SO_2_), nitrogen dioxide (NO_2_), carbon monoxide (CO), and ozone (O_3_) were estimated using high-resolution spatiotemporal data from the China High Air Pollutants dataset. We employed conditional logistic regression models to estimate associations between pollutant exposures and SMDs onset, controlling for temperature and humidity. Stratified analyses were performed to identify potentially vulnerable subpopulations.

**Results:**

Each interquartile range increase in exposure to PM_2.5_ (23.1 μg/m^3^), CO (0.51 mg/m^3^), NO_2_ (11.9 μg/m^3^), PM_10_ (57.5 μg/m^3^), and SO_2_ (19.1 μg/m^3^) was associated with increased odds of SMDs onset: 3.61% (95% CI: 1.53%-5.74%), 16.54% (95% CI: 12.26%-20.99%), 9.61% (95% CI: 6.34%-13.00%), 2.15% (95% CI: 0.90%-3.40%), and 28.04% (95% CI: 22.16%-34.20%), respectively. Exposure–response relationships displayed positive trends for all significant pollutants. Effect estimates were generally stronger among females, elderly individuals (≥ 65 years), those with higher socioeconomic status, and during warm seasons (May–October). Associations remained robust in two-pollutant models and various sensitivity analyses.

**Conclusions:**

Short-term exposure to multiple air pollutants is positively associated with SMDs onset, with differential vulnerability across population subgroups. These results suggest that air pollution may represent an important modifiable environmental risk factor for SMDs, particularly in regions with elevated pollution levels.

**Supplementary Information:**

The online version contains supplementary material available at 10.1186/s12889-025-25906-z.

## Introduction

Severe mental disorders (SMDs), such as schizophrenia, bipolar disorder, and major depression, are characterized by significant alterations in cognition, emotion regulation, and behavior that substantially impair daily functioning [1]. Mental disorders constitute a major global public health challenge, ranking among the top ten leading causes of disease burden worldwide in 2021, accounting for approximately 1.1 billion prevalence counts (14.4% of the total) and 155.4 million disability-adjusted life-years (5.4% of the total) [2]. China bears a disproportionate share of this burden, contributing approximately 15.9% of global cases and 14.9% of global disability, representing the second-largest number of cases worldwide [3]. Given the considerable socioeconomic impact and limited healthcare resources, severe mental disorders have become a priority focus in China's public health initiatives [4, 5].

Growing evidence identifies ambient air pollution as a significant environmental risk factor for mental health outcomes [6–8]. Epidemiological studies have demonstrated positive associations between air pollution exposure and various mental disorders, including schizophrenia [9], depression [10, 11], and anxiety [12, 13]. These associations are supported by toxicological studies suggesting that air pollutants may induce neuroinflammation, oxidative stress, and hypothalamic–pituitary–adrenal axis dysregulation, potentially affecting neurodevelopment and precipitating or exacerbating psychiatric symptoms [14].

While substantial evidence supports the association between long-term air pollution exposure and mental disorders, research examining short-term exposure effects remains limited and inconsistent. Existing multi-city studies in China, the U.S., and elsewhere have been instrumental in establishing initial evidence but often face methodological constraints that limit the precision and generalizability of their findings. These include limited statistical power to examine vulnerable subpopulations due to modest sample sizes, and exposure misclassification from reliance on sparse monitoring networks with low spatial resolution, which fails to capture fine-scale variations in personal exposure. Furthermore, few studies have specifically investigated these associations in regions with particularly severe air pollution and unique geographical conditions, such as northwestern China, where frequent pollution episodes and dust storms may present distinct risk profiles. Additionally, comprehensive research on severe mental disorders as a collective category is notably lacking, particularly in the Chinese population [15].

To fill these research gaps, we investigate the associations between short-term exposure to six major air pollutants and the onset of severe mental disorders using high-resolution exposure data and a case-crossover design in Gansu Province, China, from 2013 to 2020. Our study's large, population-based sample of 49,707 SMDs cases provides substantial statistical power to conduct robust stratification analyses and identify potentially vulnerable subgroups. Moreover, the high spatial resolution (1–10 km) of our exposure data minimizes misclassification bias, enabling a more precise estimation of the acute effects of air pollution on SMDs in an understudied, high-exposure setting.

## Methods

### Study population

Using the Mental Health System of the Gansu Provincial Centre for Disease Control and Prevention, we included individuals who were diagnosed with SMDs in Gansu province, China, between January 1, 2013, and December 30, 2020. This comprehensive surveillance system covers the entire population of Gansu province during the study period.

Gansu province is located in northwestern China, spanning approximately 425,800 km^2^ with an east–west extension of 1480 km and a north–south span of 1132 km. In 2020, the population was approximately 25 million (representing about 1.8% of China's total population), with an estimated population density of 59 people/km^2^ [16]. For each case, we extracted demographic information including sex, age at onset, race, residential address, and date of onset.

### Outcomes

The primary outcome was the onset of severe mental disorders as defined according to the International Statistical Classification of Diseases and Related Health Problems Tenth Revision (ICD-10), covering "schizophrenia, schizotypal, and delusional disorders", "paranoid psychosis", "bipolar disorder, psychotic disorder due to epilepsy", and "mental retardation with mental disorders" (ICD-10 codes: F00-F99) [17]. For subgroup analyses, we focused on three major diagnostic categories: "schizophrenia, schizotypal, and delusional disorders" (ICD-10 codes: F20-F29), "mood disorders" (ICD-10 codes: F30-F39), and "other mental disorders", as the first two categories represented 77.9% of all identified SMDs cases in our study population.

Date of onset for each case was precisely documented through two complementary channels: (1) mandatory reporting by community healthcare centers, which are required by national regulations to identify and register all SMDs cases within their jurisdictions; and (2) hospital-based diagnosis reporting, where all medical institutions are required to report newly diagnosed SMDs cases to the central Mental Health System. This dual-source reporting system ensures comprehensive case ascertainment throughout the province. To prevent duplicate counting from the dual-source reporting system, we utilized patients' unique national identity card numbers to identify and merge records. For cases reported by both community health centers and hospitals, only the record with the earliest documented onset date was retained in the final analytic dataset.

### Study design

We employed a time-stratified case-crossover design to evaluate the associations between air pollution and SMDs onset. This design is particularly advantageous for studying acute effects of environmental exposures as each case serves as their own control, effectively controlling for time-invariant individual confounders such as genetic factors, socioeconomic status, and lifestyle characteristics [18]. The case day was defined as the date of SMDs onset. Control days were selected from the same year, month, and day of the week as the case day, resulting in 3–4 control days matched to each case day. This time-stratified approach controls for long-term trends, seasonality, and day-of-week effects. By comparing exposure levels between case and control days, we estimated the relative risk associated with short-term air pollution exposure.

### Exposure assessment

We assessed exposure to six major air pollutants: particulate matter ≤ 2.5 μm in aerodynamic diameter (PM_2.5_), particulate matter ≤ 10 μm in aerodynamic diameter (PM_10_), sulfur dioxide (SO_2_), nitrogen dioxide (NO_2_), carbon monoxide (CO), and ozone (O_3_). Daily average pollutant concentrations were obtained from the China High Air Pollutants (CHAP) dataset, which provides high-resolution spatial data: 1 km resolution for PM_2.5_, PM_10_, and O_3_, and 10 km resolution for SO_2_, NO_2_, and CO [19–24]. The CHAP dataset integrates comprehensive, high-resolution, and high-quality ground-level air pollution data from diverse sources (for example, ground observations, satellite remote sensing, model simulations, and atmospheric reanalysis), processed using artificial intelligence [25]. The cross-validation coefficient of determination (CV-R^2^) ranging from 0.84 to 0.93 for PM_2.5_, PM_10_, O_3_, NO_2_, SO_2_, and CO, respectively [19–24]. To assign pollutant exposures, we first converted the residential address of each case into geographic coordinates (latitude and longitude) using the Baidu Maps Geocoding API (Application Programming Interface, available at: https://lbsyun.baidu.com/). The daily concentration for each pollutant was then extracted from the CHAP grid cell in which the individual's geocoded residence was located.

### Covariates

Daily meteorological data, including temperature and relative humidity were extracted from the fifth generation of European ReAnalysis (ERA5)-Land dataset, which had a spatial resolution of around 10 km × 10 km [26]. These data were assigned to each individual by extracting the values from the grid cell encompassing the geographic coordinates of their residence and were incorporated into all statistical models to control for potential confounding effects. Due to the case-crossover design, individual-level variables such as sex, age, race, genetics, and lifestyle factors were inherently controlled for, as these characteristics remain constant between case and control days. To account for potential holiday effects, data on public holidays were collected from the official announcements issued by the General Office of the State Council of China from 2013 to 2020 and included in the models as a binary covariate (holiday vs. non-holiday).

### Statistical Analysis

The relationships between air pollutants and meteorological conditions were measured using the Spearman correlation coefficients. Conditional logistic regression models were used to explore the associations of air pollutants with SMDs onset cross various exposure windows. Results are presented as percentage changes in odds ratios (ORs) of SMDs onset per interquartile range (IQR) increase in each pollutant ([OR-1] × 100%) with corresponding 95% confidence intervals (CIs).

To identify the most relevant exposure windows, we examined single-day lag effects from lag 0 to lag 6 and moving averages over multiple days from lag 01 to lag 06. For each pollutant, the lag period demonstrating the strongest association was selected for primary analyses. All models included natural cubic spline functions of daily average temperature (degrees of freedom [*df*] = 6) and relative humidity (*df*= 3) to adjust for meteorological conditions [27]. To explore potential non-linear exposure–response relationships, we applied a 3 *df* natural cubic spline transformation to each air pollutant in the main lag period, with nonlinearity evaluated using likelihood ratio tests comparing linear and spline models.

We conducted subgroup analyses stratified by sex (male, female), age (< 65 or ≥ 65 years), season (warm: May to October and cold: November to April), and disease subtype (“schizophrenia, schizotypal, and delusional disorders”, “mood disorders”, and “other mental disorders”) to identify potentially susceptible populations and examine possible effect modifications. The difference across each stratification variable was calculated using 2-sample z tests, with the point estimates of both stratifications (β = ln odds ratio) and the standard errors (SEs) [28].

$$z= \frac{{\beta }_{1}-{\beta }_{2}}{\sqrt{{SE}_{1}^{2}+{SE}_{2}^{2}}}$$

Sensitivity analyses were conducted to test the robustness of our results. Prior to these analyses, and specifically for the two-pollutant models, collinearity between pollutants was assessed using Spearman correlation coefficients and Variance Inflation Factors (VIF); all VIF values were below 3.0, indicating that multicollinearity was not a critical concern (see Supplementary Table S2). The sensitivity analyses included: 1) two-pollutant models controlling for co-pollutants; 2) restricting the analyses to cases diagnosed with “schizophrenia, schizotypal, and delusional disorders” (ICD-10 code: F20-F29); 3) restricting the analyses to Han race; 4) adjusting for temperature using a *df* of 3.

All analyses were performed using R version 4.4.2. All *P* values were reported as two-sided values, and a *P* value less than 0.05 was regarded as statistically significant.

## Results

### Descriptive statistics

During the 8-year study period (2013–2020), 49,707 SMDs cases were identified in Gansu Province, which included 30,983 cases (62.3%) of schizophrenia, schizotypal, and delusional disorders, 7,760 cases (15.6%) of mood disorders, and 10,964 cases (22.1%) of other mental disorders (Table 1). Of the cases, 50.4% were male and 91.7% were of the Han race. The mean age was 40.7 years, with 91.8% of individuals aged under 65 years. In terms of socioeconomic status, the majority of cases (60.1%) were classified as having low socioeconomic status. Case distribution was slightly higher during the warm season (May–October, 50.9%) compared to the cool season (November–April, 49.1%). The geographical distribution of cases is shown in Figure S1, with higher case densities represented by darker clusters. Our case-crossover design yielded 198,828 control days matched to the 49,707 case days.

**Table 1:** Baseline characteristics of study population

Baseline Characteristic	Value
Total Number, n	49,707
Case days, n	49,707
Control days, n	198,828
Sex, n (%)	
Male	25,054 (50.4)
Female	24,653 (49.6)
Age	
Mean (SD), yr	40.7 (17.0)
Median (IQR), yr	42.2 (25.3)
Age, n (%)	
< 65 yr	45,643 (91.8)
≥ 65 yr	4,064 (8.2)
Race, n (%)	
Han	45,576 (91.7)
Other	4,131 (8.3)
Diagnostic subgroups, n (%)	
Schizophrenia, schizotypal, and delusional disorders	30,983 (62.3)
Mood disorders	7,760 (15.6)
Other mental disorders	10,964 (22.1)
Season^*^, n (%)	
Cold season	24,386 (49.1)
Warm season	25,321 (50.9)
Socioeconomic status, n (%)	
High	19,841 (39.9)
Low	29,866 (60.1)

^*^Cold season, November to April; Warm season, May to October

*Abbreviations*: *SD* Standard deviation, *IQR* Interquartile range

Table 2 presents the distribution of air pollutants and meteorological conditions during the study period. Mean concentrations were 37.9 μg/m^3^ for PM_2.5_, 92.2 μg/m^3^ for PM_10_, 23.1 μg/m^3^ for SO_2_, 25.1 μg/m^3^ for NO_2_, 1.0 mg/m^3^ for CO, and 94.7 μg/m^3^ for O_3_. The correlation analysis (Table S1) revealed moderate to strong positive correlations among PM_2.5_, CO, NO_2_, PM_10_, and SO_2_. In contrast, O_3_ exposure was negatively associated with other pollutants. Temperature was positively associated with O_3_ and negatively associated with air pollutants, while relative humidity was negatively associated with all air pollutants.

**Table 2:** Distribution of air pollutants and meteorological conditions during case and control days in Gansu Province, China, 2013–2020

Variable	Mean	SD	Min	P_25_	Median	P_75_	Max
Air pollutant							
PM_2.5_, μg/m^3^	37.9	20.0	2.5	24.3	33.9	47.4	568.1
CO, mg/m^3^	1.0	0.4	0.03	0.6	0.9	1.1	11.3
NO_2_, μg/m^3^	25.1	12.0	1.5	17.7	22.5	29.6	208.8
O_3_, μg/m^3^	94.7	25.4	5.9	74.4	93.5	112.7	619.6
PM_10_, μg/m^3^	92.2	68.4	7.9	54.2	79.1	111.7	2,508.7
SO_2_, μg/m^3^	23.1	18.5	1.1	10.5	16.1	29.6	610.2
Meteorological condition							
Relative humidity, %	55.2	21.1	3.3	38.7	55.7	72.0	103.5
Temperature, ℃	7.7	10.3	−29.5	−0.8	8.7	16.0	38.1

*Abbreviations SD* Standard deviation, *P*_*25*_ The 25th percentile, *Median* The 50th percentile, *P*_*75*_ The 75th percentile, *PM*_*2.5*_ Particulate matter with an aerodynamic diameter ≤ 2.5 μm, *CO* Carbon monoxide, *NO*_*2*_ Nitrogen dioxide, *O*_*3*_ Ozone, *PM*_*10*_ Particulate matter with an aerodynamic diameter ≤ 10 μm, *SO*_*2*_ Sulfur dioxide

### Associations of air pollutants with SMDs

**Fig. 1:**
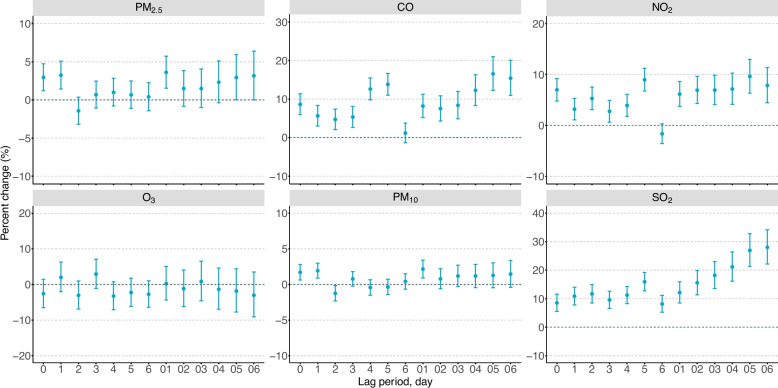
Percentage changes in ORs and 95% confidence intervals for onset of severe mental disorders associated with each interquartile range increase of exposures to PM_2.5_ (interquartile range, 23.1 μg/m^3^), PM_10_ (57.5 μg/m^3^), SO_2_ (19.1 μg/m^3^), NO_2_ (11.9 μg/m^3^), CO (0.51 mg/m^3^), and O_3_ (38.3 μg/m^3^) with different lag periods. Abbreviations: PM_2.5_, particulate matter with an aerodynamic diameter ≤ 2.5 μm; CO, carbon monoxide; NO_2_, nitrogen dioxide; O_3_, ozone; PM_10_, particulate matter with an aerodynamic diameter ≤ 10 μm; SO_2_, sulfur dioxide

Figure 1 shows the lag-specific associations between air pollutants and SMDs onset. Significant positive associations were observed for PM_2.5_ (lag0, lag1, lag01, lag05-lag06), CO (lag0-lag5, lag01-lag06), NO_2_ (lag0-lag5, lag01-lag06), PM_10_ (lag0, lag1, lag01) and SO_2_ (lag0-lag6, lag01-lag06), with the strongest effects generally observed at cumulative exposure windows. For subsequent analyses, we selected the exposure windows demonstrating the strongest associations: lag01 for PM_2.5_, lag05 for CO, lag05 for NO_2_, lag01 for PM_10,_ and lag06 for SO_2_. Each IQR increase in pollutant concentration was associated with significantly increased odds of SMDs onset: 3.61% (95% CI: 1.53%−5.74%) for PM_2.5_, 16.54% (95% CI: 12.26%−20.99%) for CO, 9.61% (95% CI: 6.34%−13.00%) for NO_2_, 2.15% (95% CI: 0.90%−3.40%) for PM_10_, and 28.04% (95% CI: 22.16%−34.20%) for SO_2_.

**Fig. 2:**
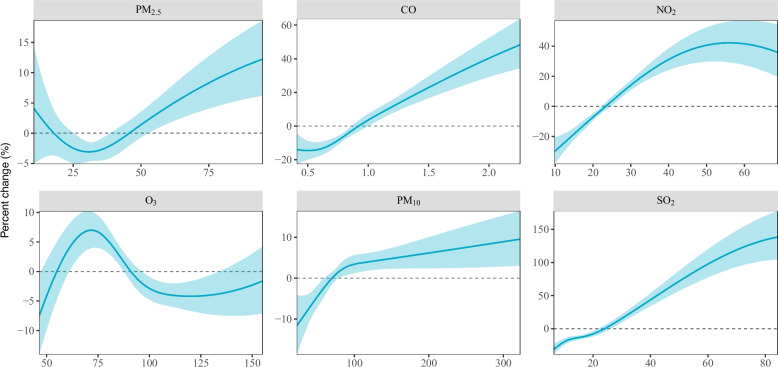
Exposure–response curves between exposures to PM_2.5_ (lag01), CO (lag05), NO_2_ (lag05), O_3_ (lag3), PM_10_ (lag01), SO_2_ (lag06), and onset of severe mental disorders. Abbreviations: PM_2.5_, particulate matter with an aerodynamic diameter ≤ 2.5 μm; CO, carbon monoxide; NO_2_, nitrogen dioxide; O_3_, ozone; PM_10_, particulate matter with an aerodynamic diameter ≤ 10 μm; SO_2_, sulfur dioxide

The concentration–response curves for each pollutant and SMDs onset are presented in Fig. 2. We observed monotonically increasing relationships between SMDs risk and concentrations of CO, PM_10_, and SO_2_ across their respective distribution ranges. For PM_2.5_, the risk increased monotonically at concentrations above 29.4 μg/m^3^, while for NO_2_, a similar positive trend was observed at concentrations below 56.1 μg/m^3^. Likelihood ratio tests confirmed significant non-linearity in the exposure–response relationships for PM_2.5_ and NO_2_ (*P* < 0.05), while relationships for CO, PM_10_, and SO_2_ did not significantly deviate from linearity.

### Stratified analyses

Stratified analyses revealed distinct vulnerability patterns. Females demonstrated significantly stronger associations with PM₁₀ exposure compared to males. Elderly individuals (≥ 65 years) showed markedly elevated susceptibility to PM_2.5_, NO₂, and SO₂. Seasonal variations were evident, with enhanced warm-season effects for PM_2.5_, CO, and NO₂, while SO₂ exhibited stronger cold-season associations. Analysis by diagnostic categories showed SO₂ consistently associated with increased risk across all subtypes, though PM₁₀ was not significantly associated with schizophrenia spectrum disorders. Notably, higher socioeconomic status predicted greater susceptibility to PM_2.5_, NO₂, and SO₂. (Table 3).

**Table 3:** Percent changes in ORs and 95% confidence intervals for onset of severe mental disorders associated with each IQR increase of exposures to PM_2.5_ (lag01), CO (lag05), NO_2_ (lag 05), PM_10_ (lag01), and SO_2_ (lag06) stratified by sex, age, season, disease subtype and socioeconomic status

Variable	Percent change (95% CI)				
	PM_2.5_	CO	NO_2_	PM_10_	SO_2_
Sex					
Male	1.81 (−1.13,4.83)	**19.06 (12.88,25.57)**	**10.68 (5.96,15.61)**	0.05 (−1.71,1.84) *	**34.17 (25.42,43.54)**
Female	**5.41 (2.48,8.43)**	**14.09 (8.24,20.25)**	**8.61 (4.10,13.32)**	4.20 (2.44,5.98) *	**22.38 (14.57,30.73)**
Age					
< 65	2.95 (0.79,5.16) *	**15.65 (11.22,20.26)**	8.49 (5.07,12.01) *	**2.51 (1.21,3.83)**	25.74 (19.77,32.00) *
≥ 65	11.06 (3.50,19.16) *	**25.63 (10.22,43.18)**	20.32 (9.05,32.75) *	−1.55 (−5.93,3.04)	61.37 (34.51,93.59) *
Season					
Warm	**4.45 (1.55,7.45)**	43.14 (32.05,55.16) *	23.53 (15.01,32.69) *	1.82 (−0.03,3.70)	**21.92 (9.21,36.11)**
Cold	1.41 (−1.52,4.44)	14.25 (9.43,19.28) *	8.26 (4.66,11.97) *	**1.92 (0.24,3.63)**	**37.40 (30.24,44.97)**
Disease subtype					
Schizophrenia, schizotypal, and delusional disorders	**3.50 (0.90,6.17)**	18.00 (12.68,23.57) *	**9.73 (5.68,13.94)**	1.27 (−0.31,2.87)	**27.11 (19.92,34.73)**
Mood disorders	4.73 (−0.53,10.26)	23.90 (11.84,37.27) *	**13.18 (4.08,23.08)**	**4.78 (1.65,8.00)**	**36.87 (21.23,54.52)**
Other mental disorders	2.96 (−1.50,7.62)	7.02 (−1.44,16.21) *	6.75 (−0.02,13.98)	**2.61 (0.00,5.29)**	**23.31 (10.91,37.10)**
Socioeconomic status					
Low	−0.10 (−2.70,2.58) *	12.17 (6.90,17.69) *	4.78 (0.86,8.86) *	1.43 (−0.11,2.99)	19.37 (12.67,26.47) *
High	9.39 (5.95,12.95) *	22.84 (15.69,30.43) *	17.82 (12.03,23.91) *	3.59 (1.48,5.74)	43.88 (32.88,55.80) *

Statistically significant associations are shown in bold

^*^differences across strata are statistically significant

*Abbreviations*: *PM*_*2.5*_ Particulate matter with an aerodynamic diameter ≤ 2.5 μm, *CO* Carbon monoxide, *NO*_*2*_ Nitrogen dioxide, *O*_*3*_ Ozone, *PM*_*10*_ Particulate matter with an aerodynamic diameter ≤ 10 μm, *SO*_*2*_ Sulfur dioxide

### Sensitivity analyses

Two-pollutant models were conducted to test the robustness of our findings (Table 4). The associations between each pollutant and SMDs onset remained statistically significant after adjustment for co-pollutants, with the exception of NO_2_, which became non-significant when adjusted for other pollutants. To prevent multicollinearity issues, we did not include PM_2.5_ and PM_10_ in the same model. Additional sensitivity analyses restricting the study population to Han ethnicity or to cases diagnosed with schizophrenia spectrum disorders yielded results consistent with our primary findings (Figures S2-S3). Similarly, using an alternative approach to control for temperature (*df* = 3 instead of *df* = 6) did not materially change the observed associations (Figure S4), further supporting the robustness of our findings.

**Table 4:** Percent changes in ORs and 95% confidence intervals for onset of severe mental disorders associated with each IQR increase of exposures to PM_2.5_ (lag01), CO (lag05), NO_2_ (lag 05), PM_10_ (lag01), and SO_2_ (lag06) estimated by Single- and Two-pollutant Models

Models	Percent change (95% CI)				
	PM_2.5_	CO	NO_2_	PM_10_	SO_2_
Single	3.61 (1.53,5.74)	16.54 (12.26,20.99)	9.62 (6.34,13.00)	2.15 (0.90,3.41)	28.04 (22.16,34.20)
Adjusted for PM_2.5_	—	15.98 (11.50,20.65)	9.34 (5.95,12.84)	—	27.15 (20.99,33.62)
Adjusted for CO	2.42 (0.29,4.6)	—	3.19 (−0.55,7.06)	2.06 (0.82,3.32)	23.82 (17.62,30.36)
Adjusted for NO_2_	2.71 (0.6,4.87)	14.01 (8.94,19.31)	—	2.20 (0.95,3.46)	27.76 (21.29,34.57)
Adjusted for PM_10_	10.37 (6.45,14.44)	16.43 (12.15,20.87)	9.57 (6.29,12.95)	—	27.89 (22.02,34.05)
Adjusted for SO_2_	2.65 (0.56,4.79)	8.73 (4.33,13.31)	2.75 (−0.65,6.26)	2.07 (0.82,3.33)	—
Adjusted for O_3_	3.57 (1.49,5.70)	15.77 (11.51,20.20)	9.48 (6.20,12.86)	2.11 (0.86,3.36)	26.78 (20.95,32.89)

*Abbreviations*: *PM*_*2.5*_ Particulate matter with an aerodynamic diameter ≤ 2.5 μm, *CO* Carbon monoxide, *NO*_*2*_ Nitrogen dioxide, *O*_*3*_ Ozone, *PM*_*10*_ Particulate matter with an aerodynamic diameter ≤ 10 μm, *SO*_*2*_ Sulfur dioxide

## Discussion

In this large case-crossover study involving 49,707 individuals with SMDs in northwestern China, we found positive associations between short-term exposure to multiple air pollutants and SMDs onset. Specifically, each IQR increase in PM_2.5_, CO, NO_2_, PM_10_, and SO_2_ was associated with increased odds of SMDs onset ranging from 2.15% to 28.04%, with exposure–response relationships exhibiting positive trends. Our analyses further revealed potentially vulnerable subgroups, with females demonstrating greater susceptibility to PM_10_, elderly individuals showing stronger associations with PM_2.5_, NO_2_, and SO_2_, individuals with higher socioeconomic status exhibiting enhanced vulnerability to multiple pollutants including PM_2.5_, CO, NO_2_, and SO_2_, and warm-season exposures exhibiting more pronounced effects for several pollutants.

This study represents the first comprehensive investigation of short-term air pollution effects on severe mental disorders in northwestern China, employing high-resolution exposure data, a large population-based sample, and rigorous methodology to address critical knowledge gaps in environmental psychiatry. Our findings extend the current understanding of air pollution's relationship with mental health in several important ways. While previous studies have examined associations between air pollution and specific psychiatric conditions or healthcare utilization, our research uniquely addresses SMDs as a broader category with significant public health implications. Several multi-city studies in China reported positive associations between air pollutants and hospital admissions for mental disorders, but with substantially different exposure assessment methodology and geographical context [29–31]. Similarly, research in the United States found positive associations between PM_2.5_ and NO_2_exposure and psychiatric hospital admissions, though with different effect magnitudes and population characteristics [32]. The divergence between our findings and those reported in some European studies, which found limited associations except for O_3_, likely reflects regional differences in pollution characteristics, healthcare systems, and population susceptibilities—underscoring the importance of context-specific environmental health research [33].

Notably, the magnitude of the SO₂ association observed in our study was larger than estimates from some prior studies. This may be attributable to several factors specific to our study context. First, the distinct pollution composition in northwestern China, characterized by substantial emissions from coal combustion for industry and residential heating—a primary source of SO₂—may result in a pollutant mixture with enhanced neurotoxic potential [34]. Second, differences in outcome definition are crucial to consider. While many previous studies relied on hospital admissions data that capture exacerbations of pre-existing conditions, our outcome captures the initial onset of SMDs through a comprehensive surveillance system. The initial manifestation of severe mental disorders likely represents a period of particular biological vulnerability, where environmental triggers may have more pronounced effects compared to subsequent exacerbations in established disease [35]. Additionally, regional variations in healthcare seeking behavior and diagnostic practices, combined with our study's unique setting in an understudied region with high pollution levels, may contribute to the observed effect size differences. This finding underscores that the magnitude of air pollution health risks can vary significantly by regional pollution profile and the specific health endpoint examined, with the initial onset of disease representing a critical window for intervention.

While the precise mechanisms linking short-term air pollution exposure to SMDs onset remain incompletely understood, several acute biological pathways offer plausible explanations for our observed associations. Inhaled pollutants can rapidly induce systemic inflammation and oxidative stress, leading to increased circulating pro-inflammatory cytokines (e.g., IL-6, TNF-α) and reactive oxygen species [36]. These can, in turn, initiate acute neuroinflammation by activating microglia and compromise blood–brain barrier function within hours to days [37]. Furthermore, air pollution exposure may act as a physiological stressor, leading to the acute activation of the hypothalamic–pituitary–adrenal (HPA) axis and altering cortisol dynamics, which is known to influence mood and psychosis [38]. In susceptible individuals, these rapid physiological perturbations could potentially lower the threshold for psychiatric symptom manifestation, precipitating the acute onset of SMDs during high-pollution episodes.

The observed vulnerability patterns in our study offer important insights for targeted public health interventions. The enhanced susceptibility among females to particulate matter aligns with emerging evidence of sex-based differences in air pollution responses, potentially attributable to physiological differences in immune response, hormonal factors, or differential exposure patterns [39–41]. The stronger associations observed among elderly individuals for multiple pollutants suggest age-related vulnerability mechanisms, possibly related to decreased physiological reserve, compromised blood–brain barrier integrity, or reduced antioxidant capacity with advancing age [42, 43]. The counterintuitive finding of greater susceptibility in higher socioeconomic groups may be explained by several factors: their potentially distinct exposure profiles, such as longer commutes in traffic-congested urban areas or greater participation in outdoor activities; differential healthcare-seeking behaviors that could lead to more complete case ascertainment. Seasonal variations in effect estimates, with stronger associations during warmer months, may reflect seasonal differences in activity patterns, ventilation behaviors, or photochemical pollutant formation [44]. These heterogeneous effects highlight the importance of identifying vulnerable subpopulations for targeted protection measures, particularly as climate change may exacerbate both air pollution and temperature extremes in coming decades.

Our study has several strengths that enhance the reliability and significance of the findings. First, the time-stratified case-crossover design effectively controls for individual-level confounders and time trends, providing robust estimates of acute exposure effects. Second, our use of high-resolution air pollution data (1–10 km) based on multiple data sources represents a substantial improvement over previous studies relying on sparse monitoring networks, significantly reducing exposure misclassification. Third, the large sample size drawn from comprehensive provincial surveillance data provides sufficient statistical power for detecting subtle associations and conducting meaningful subgroup analyses. Fourth, the study setting in Gansu Province—characterized by severe air pollution episodes and unique geographical conditions—offers valuable insights into pollution-health relationships in a previously understudied high-exposure setting [15]. Finally, our extensive sensitivity analyses demonstrate the robustness of our findings across alternative model specifications and subpopulations.

Several limitations should be considered when interpreting our results. First, despite using high-resolution spatial data and point-level geocoding of residential addresses, we could not account for individual time-activity patterns or indoor air pollution exposure, potentially introducing some exposure misclassification [45]. For instance, our assessment does not capture exposures experienced at workplaces, during commutes, or indoors. However, the case-crossover design, which compares exposures within individuals across time rather than between individuals, likely minimizes the impact of this limitation. Furthermore, the use of meteorological data at a 10 km resolution, while standard in large-scale epidemiological studies, may not capture microclimate variations (e.g., urban heat islands) and could introduce some residual confounding, despite our efforts to control for these factors in the models. Second, while we controlled for temperature and humidity, other time-varying factors such as viral epidemics, social events, or healthcare access fluctuations might introduce residual confounding. Third, the strong correlations among pollutants present challenges in disentangling the independent effects of individual pollutants, although our two-pollutant models suggest persistent associations after mutual adjustment. Fourth, while we observed consistent associations across diagnostic subgroups, differences in case ascertainment or diagnostic practices across the region could influence our findings. Finally, caution is warranted in generalizing these results to other populations with different pollution profiles, genetic backgrounds, or healthcare systems.

In conclusion, this study provides robust evidence of associations between short-term exposure to multiple air pollutants and the SMDs onset in northwestern China. Our findings suggest that air pollution may represent an important modifiable environmental risk factor for mental health, with particularly pronounced effects among vulnerable subpopulations including females, the elderly, individuals with higher socioeconomic status, and during warmer seasons. These results underscore the potential public health benefits of improved air quality for reducing the burden of severe mental disorders, especially in regions with elevated pollution levels.

## Supplementary Information

Supplementary Material 1: Table S1. Spearman correlation coefficients between air pollutants and meteorological conditions during case and control days in Gansu Province, China, 2013–2020*. Table S2. Variance Inflation Factors (VIF) for pollutants in the two-pollutant models. Figure S1. Spatial distributions of 49,707 cases’ home addresses (dots) in Gansu province, China, from 2013 to 2020. Figure S2. Percentage changes in odds ratios and 95% CI for onset of severe mental disorders associated with each interquartile range increase of exposures to PM2.5 (interquartile range, 23.1 μg/m3), PM10 (57.5 μg/m3), SO2 (19.1 μg/m3), NO2 (11.9 μg/m3), CO (0.51 mg/m3), and O3 (38.3 μg/m3) with different lag periods, restricted to Han race onset cases. Figure S3. Percentage changes in odds ratios and 95% confidence intervals for onset of severe mental disorders associated with each interquartile range increase of exposures to PM2.5 (interquartile range, 23.1 μg/m3), PM10 (57.5 μg/m3), SO2 (19.1 μg/m3), NO2 (11.9 μg/m3), CO (0.51 mg/m3), and O3 (38.3 μg/m3) with different lag periods, restricted to cases who diagnosed with “schizophrenia, schizotypal, and delusional disorders”. Figure S4. Percentage changes in odds ratios and 95% confidence intervals for onset of severe mental disorders associated with each interquartile range increase of exposures to PM2.5 (interquartile range, 23.1 μg/m3), PM10 (57.5 μg/m3), SO2 (19.1 μg/m3), NO2 (11.9 μg/m3), CO (0.51 mg/m3), and O3 (38.3 μg/m3) with different lag periods, adjusting for temperature with a *df* of 3.

## Data Availability

The datasets generated and/or analysed during this study are not publicly available, but are available from the corresponding author on reasonable request.
