# Supplementary material for: Short-term exposure to ambient air pollution and onset of severe mental disorders: a case-crossover study in Northwestern China
Source: BMC Public Health. 2025 Dec 22;26:480. doi: 10.1186/s12889-025-25906-z (PMC12874826; doi:10.1186/s12889-025-25906-z)
Supplement: Supplementary file 1 — Supplementary Material 1: Table S1. Spearman correlation coefficients between air pollutants and meteorological conditions during case and control days in Gansu Province, China, 2013–2020*. Table S2. Variance Inflation Factors (VIF) for pollutants in the two-pollutant models. Figure S1. Spatial distributions of 49,707 cases’ home addresses (dots) in Gansu province, China, from 2013 to 2020. Figure S2. Percentage changes in odds ratios and 95% CI for onset of severe mental disorders associated with each interquartile range increase of exposures to PM2.5 (interquartile range, 23.1 μg/m3), PM10 (57.5 μg/m3), SO2 (19.1 μg/m3), NO2 (11.9 μg/m3), CO (0.51 mg/m3), and O3 (38.3 μg/m3) with different lag periods, restricted to Han race onset cases. Figure S3. Percentage changes in odds ratios and 95% confidence intervals for onset of severe mental disorders associated with each interquartile range increase of exposures to PM2.5 (interquartile range, 23.1 μg/m3), PM10 (57.5 μg/m3), SO2 (19.1 μg/m3), NO2 (11.9 μg/m3), CO (0.51 mg/m3), and O3 (38.3 μg/m3) with different lag periods, restricted to cases who diagnosed with “schizophrenia, schizotypal, and delusional disorders”. Figure S4. Percentage changes in odds ratios and 95% confidence intervals for onset of severe mental disorders associated with each interquartile range increase of exposures to PM2.5 (interquartile range, 23.1 μg/m3), PM10 (57.5 μg/m3), SO2 (19.1 μg/m3), NO2 (11.9 μg/m3), CO (0.51 mg/m3), and O3 (38.3 μg/m3) with different lag periods, adjusting for temperature with a df of 3. [file 12889_2025_25906_MOESM1_ESM.docx]

**Short-Term Exposure to Ambient Air Pollution and Onset of Severe Mental Disorders: A case-crossover study in northwestern China**

Yajin Han^1,†^, Guangrui Yang^2,†^, Jinshi Wang^1^, Weimin Pan^3,*^, Xiaofeng Luo^1,*^

**Table S1.** Spearman correlation coefficients between air pollutants and meteorological conditions during case and control days in Gansu Province, China, 2013-2020^*^.

**Table S2.** Variance Inflation Factors (VIF) for pollutants in the two-pollutant models.

**Figure S1.** Spatial distributions of 49,707 cases’ home addresses (dots) in Gansu province, China, from 2013 to 2020.

**Figure S2.** Percentage changes in ORs and 95% CI for onset of severe mental disorders associated with each interquartile range increase of exposures to PM_2.5_ (interquartile range, 23.1 μg/m^3^), PM_10_ (57.5 μg/m^3^), SO_2_ (19.1 μg/m^3^), NO_2_ (11.9 μg/m^3^), CO (0.51 mg/m^3^), and O_3_ (38.3 μg/m^3^) with different lag periods, restricted to Han race onset cases.

**Figure S3.** Percentage changes in ORs and 95% confidence intervals for onset of severe mental disorders associated with each interquartile range increase of exposures to PM_2.5_ (interquartile range，23.1 μg/m^3^), PM_10_ (57.5 μg/m^3^), SO_2_ (19.1 μg/m^3^), NO_2_ (11.9 μg/m^3^), CO (0.51 mg/m^3^), and O_3_ (38.3 μg/m^3^) with different lag periods, restricted to cases who diagnosed with “schizophrenia, schizotypal, and delusional disorders”.

**Figure S4.** Percentage changes in ORs and 95% confidence intervals for onset of severe mental disorders associated with each interquartile range increase of exposures to PM_2.5_ (interquartile range, 23.1 μg/m^3^), PM_10_ (57.5 μg/m^3^), SO_2_ (19.1 μg/m^3^), NO_2_ (11.9 μg/m^3^), CO (0.51 mg/m^3^), and O_3_ (38.3 μg/m^3^) with different lag periods, adjusting for temperature with a *df* of 3.


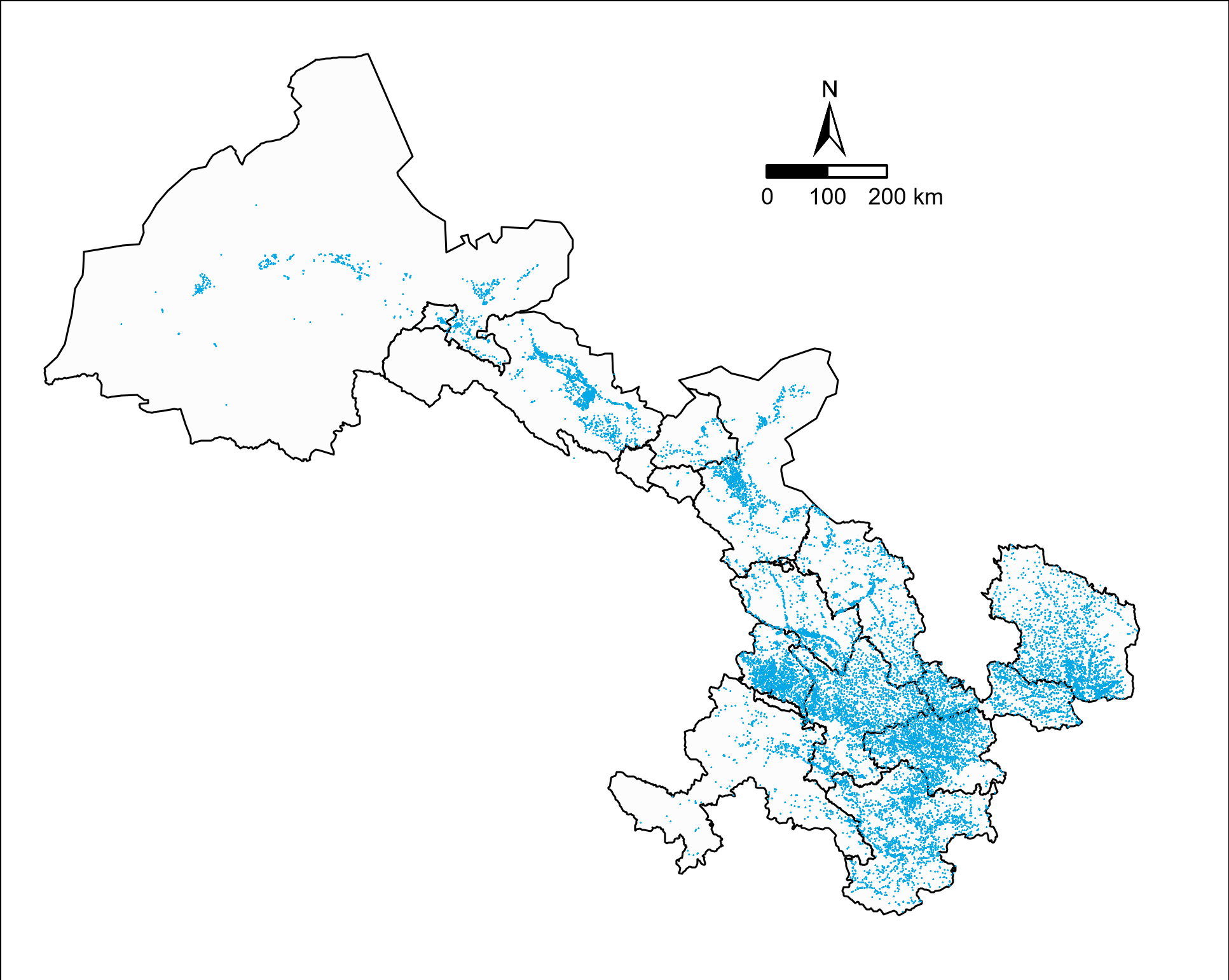


**Figure S1.** Spatial distributions of 49,707 cases’ home addresses (dots) in Gansu province, China, from 2013 to 2020. Each point indicates a case.

**Table S1.** Spearman correlation coefficients between air pollutants and meteorological conditions during case and control days in Gansu Province, China, 2013-2020^*^

|  | PM_2.5_ | CO | NO_2_ | O_3_ | PM_10_ | SO_2_ | Relative humidity |
| --- | --- | --- | --- | --- | --- | --- | --- |
| CO | 0.67 | — | — | — | — | — | — |
| NO_2_ | 0.51 | 0.61 | — | — | — | — | — |
| O_3_ | -0.45 | -0.54 | -0.37 | — | — | — | — |
| PM_10_ | 0.87 | 0.52 | 0.41 | -0.22 | — | — | — |
| SO_2_ | 0.68 | 0.74 | 0.57 | -0.48 | 0.61 | — | — |
| Relative humidity | -0.14 | -0.09 | -0.13 | -0.25 | -0.33 | -0.17 | — |
| Temperature | -0.57 | -0.50 | -0.45 | 0.67 | -0.43 | -0.56 | -0.14 |

Abbreviations: PM_2.5_, particulate matter with an aerodynamic diameter ≤2.5 μm; CO, carbon monoxide; NO_2_, nitrogen dioxide; O_3_, ozone; PM_10_, particulate matter with an aerodynamic diameter ≤10 μm; SO_2_, sulfur dioxide.

Notes^*^: All pairwise correlation coefficients were statistically significant(P<0.05)

**Table S2.** Variance Inflation Factors (VIF) for pollutants in the two-pollutant models.

|  | PM_2.5_ | CO | NO_2_ | O_3_ | PM_10_ |
| --- | --- | --- | --- | --- | --- |
| CO | 1.57 | — | — | — | — |
| NO_2_ | 1.29 | 1.82 | — | — | — |
| O_3_ | 1.22 | 1.51 | 1.22 | — | — |
| PM_10_ | 2.91 | 1.15 | 1.08 | 1.02 | — |
| SO_2_ | 1.44 | 1.97 | 1.32 | 1.25 | 1.14 |

Abbreviations: PM_2.5_, particulate matter with an aerodynamic diameter ≤2.5 μm; CO, carbon monoxide; NO_2_, nitrogen dioxide; O_3_, ozone; PM_10_, particulate matter with an aerodynamic diameter ≤10 μm; SO_2_, sulfur dioxide.

**Figure S2.** Percentage changes in ORs and 95% CI for onset of severe mental disorders associated with each interquartile range increase of exposures to PM_2.5_ (interquartile range, 23.1 μg/m^3^), PM_10_ (57.5 μg/m^3^), SO_2_ (19.1 μg/m^3^), NO_2_ (11.9 μg/m^3^), CO (0.51 mg/m^3^), and O_3_ (38.3 μg/m^3^) with different lag periods, restricted to Han race onset cases.

Abbreviations: PM_2.5_, particulate matter with an aerodynamic diameter ≤2.5 μm; CO, carbon monoxide; NO_2_, nitrogen dioxide; O_3_, ozone; PM_10_, particulate matter with an aerodynamic diameter ≤10 μm; SO_2_, sulfur dioxide.

**Figure S3.** Percentage changes in ORs and 95% confidence intervals for onset of severe mental disorders associated with each interquartile range increase of exposures to PM_2.5_ (interquartile range，23.1 μg/m^3^), PM_10_ (57.5 μg/m^3^), SO_2_ (19.1 μg/m^3^), NO_2_ (11.9 μg/m^3^), CO (0.51 mg/m^3^), and O_3_ (38.3 μg/m^3^) with different lag periods, restricted to cases who diagnosed with “schizophrenia, schizotypal, and delusional disorders”.

Abbreviations: PM_2.5_, particulate matter with an aerodynamic diameter ≤2.5 μm; CO, carbon monoxide; NO_2_, nitrogen dioxide; O_3_, ozone; PM_10_, particulate matter with an aerodynamic diameter ≤10 μm; SO_2_, sulfur dioxide.

**Figure S4.** Percentage changes in ORs and 95% confidence intervals for onset of severe mental disorders associated with each interquartile range increase of exposures to PM_2.5_ (interquartile range，23.1 μg/m^3^), PM_10_ (57.5 μg/m^3^), SO_2_ (19.1 μg/m^3^), NO_2_ (11.9 μg/m^3^), CO (0.51 mg/m^3^), and O_3_ (38.3 μg/m^3^) with different lag periods, adjusting for temperature with a *df* of 3.

Abbreviations: PM_2.5_, particulate matter with an aerodynamic diameter ≤2.5 μm; CO, carbon monoxide; NO_2_, nitrogen dioxide; O_3_, ozone; PM_10_, particulate matter with an aerodynamic diameter ≤10 μm; SO_2_, sulfur dioxide.
